# Supplementary material for: Partial Agonistic Actions of Sex Hormone Steroids on TRPM3 Function
Source: Int J Mol Sci. 2021 Dec 20;22(24):13652. doi: 10.3390/ijms222413652 (PMC8708174; doi:10.3390/ijms222413652)
Supplement: Supplementary file 1 [file ijms-22-13652-s001.zip › ijms-1524164-supplementary-3-2.pdf]

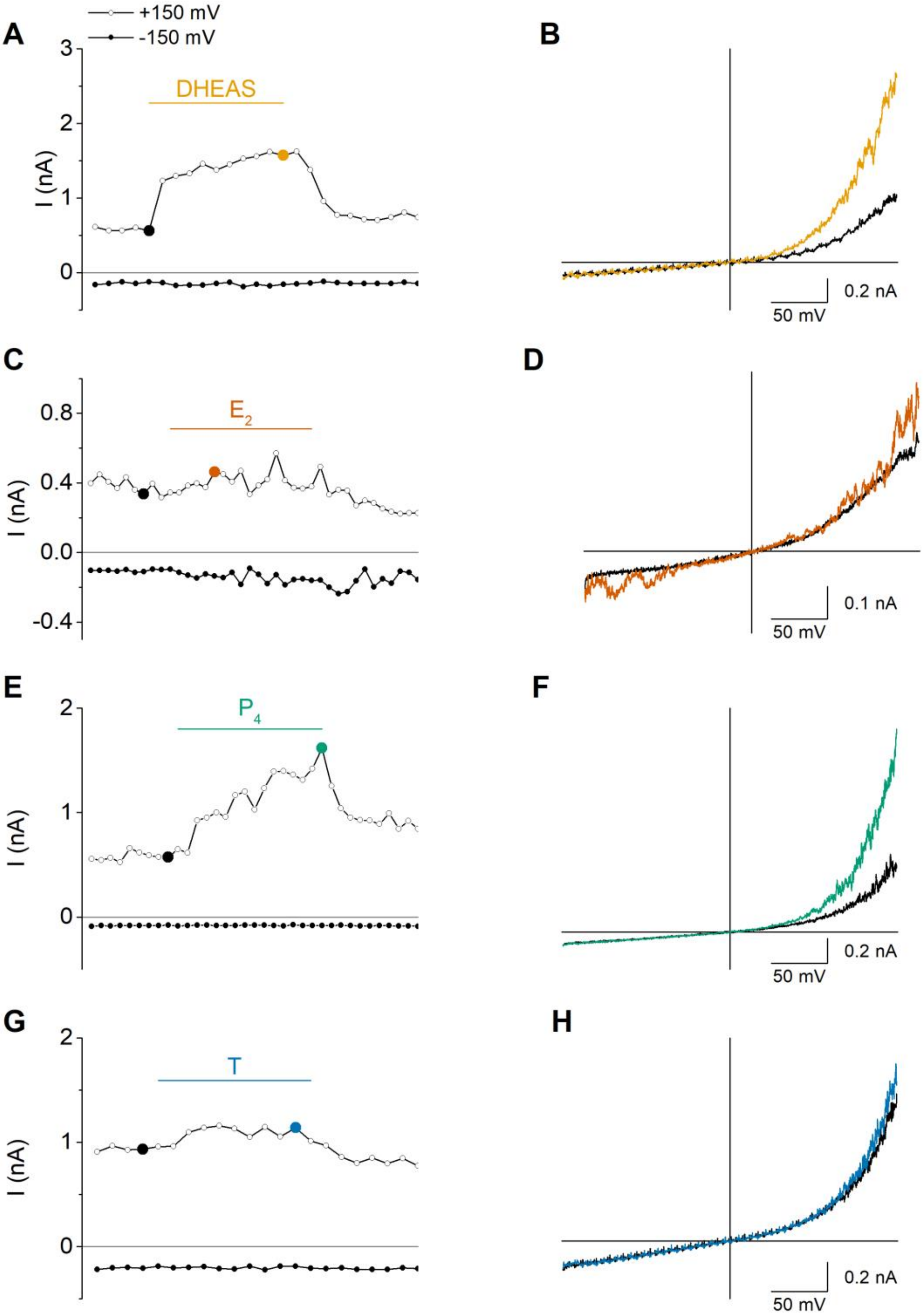

Figure S1. Patch clamp data show effect of direct application of (precursor) sex hormones on TRPM3 activity. (**A, C, E, G**) Time course of patch clamp experiments on HEK-TRPM3α2 cells, upon application of 100 μM DHEAS, E_2, P_4, and T, respectively. (**B, D, F, H**) Corresponding I-V curve of the indicated points in *(A, C, E, G)*.

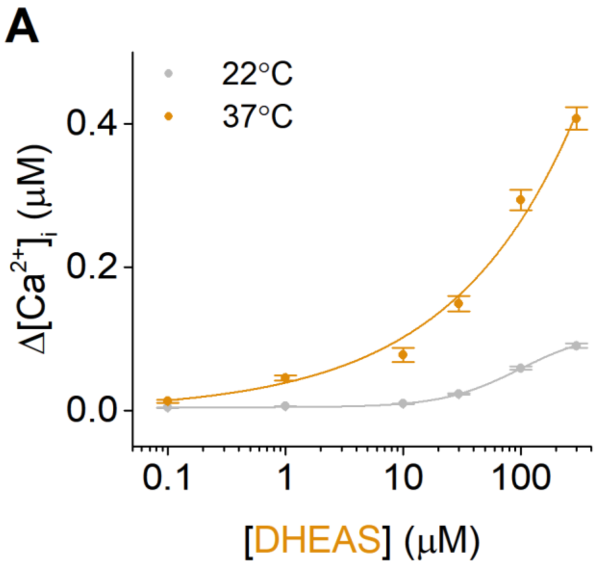

Figure S2. Effect of DHEAS on human TRPM3 isoform. (**A**) Concentration-response curves of DHEAS at 23°C (N = 4, n = 578) and 37°C (N = 3, n = 346). Data are represented as mean ± SEM.

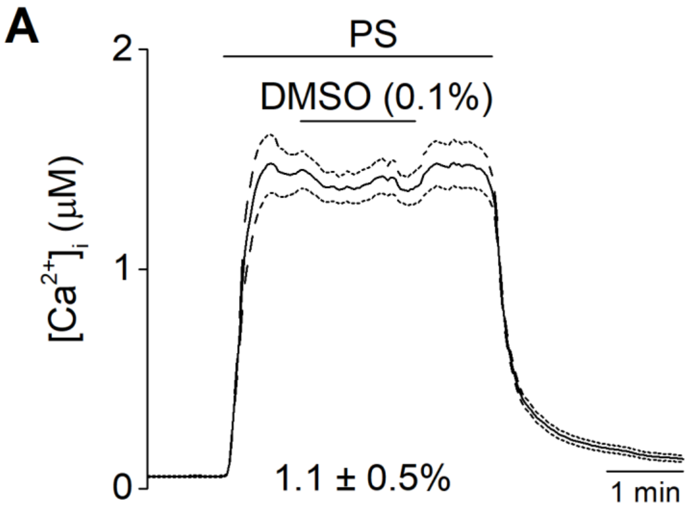

Figure S3. DMSO (0.1%) has no clear effect on PS-induced TRPM3 responses. (**A**) Time course of intracellular calcium concentrations ([Ca^{2+}]$_i$) of HEK-TRPM3 cells upon application of 40 µM PS, followed by a co-administration with 0.1% DMSO. No inhibitory effect could be detected during DMSO administration (N = 3, n = 707). Data are represented as mean ± SEM.

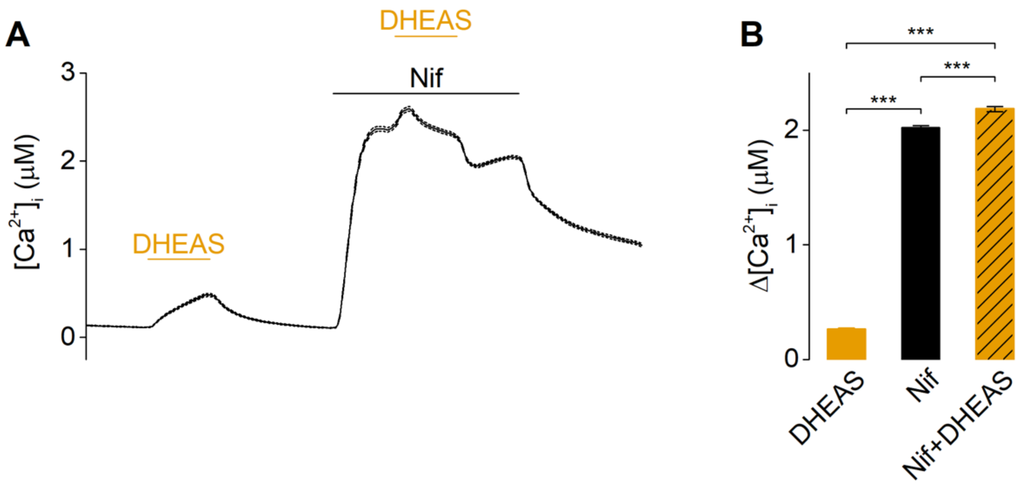

Figure S4. DHEAS does not exhibit antagonistic effects on nifedipine-induced TRPM3 activity. (**A**) Time course of intracellular calcium concentrations ($[Ca^{2+}]_i$) of HEK-TRPM3 cells upon application of 100 μM DHEAS, 50 μM nifedipine (Nif) or co-application of DHEAS and Nif (N = 3, n = 763). (**B**) Corresponding amplitudes of the calcium imaging experiment. Data are represented as mean ± SEM. Statistical analysis using a Kruskal-Wallis test and a Dunn's multiple comparisons posthoc test, p<0.05 *, p<0.01 **. P values for DHEAS/Nif, DHEAS/Nif+DHEAS and Nif/Nif+DHEAS: $p < 1*10^{-15}$.

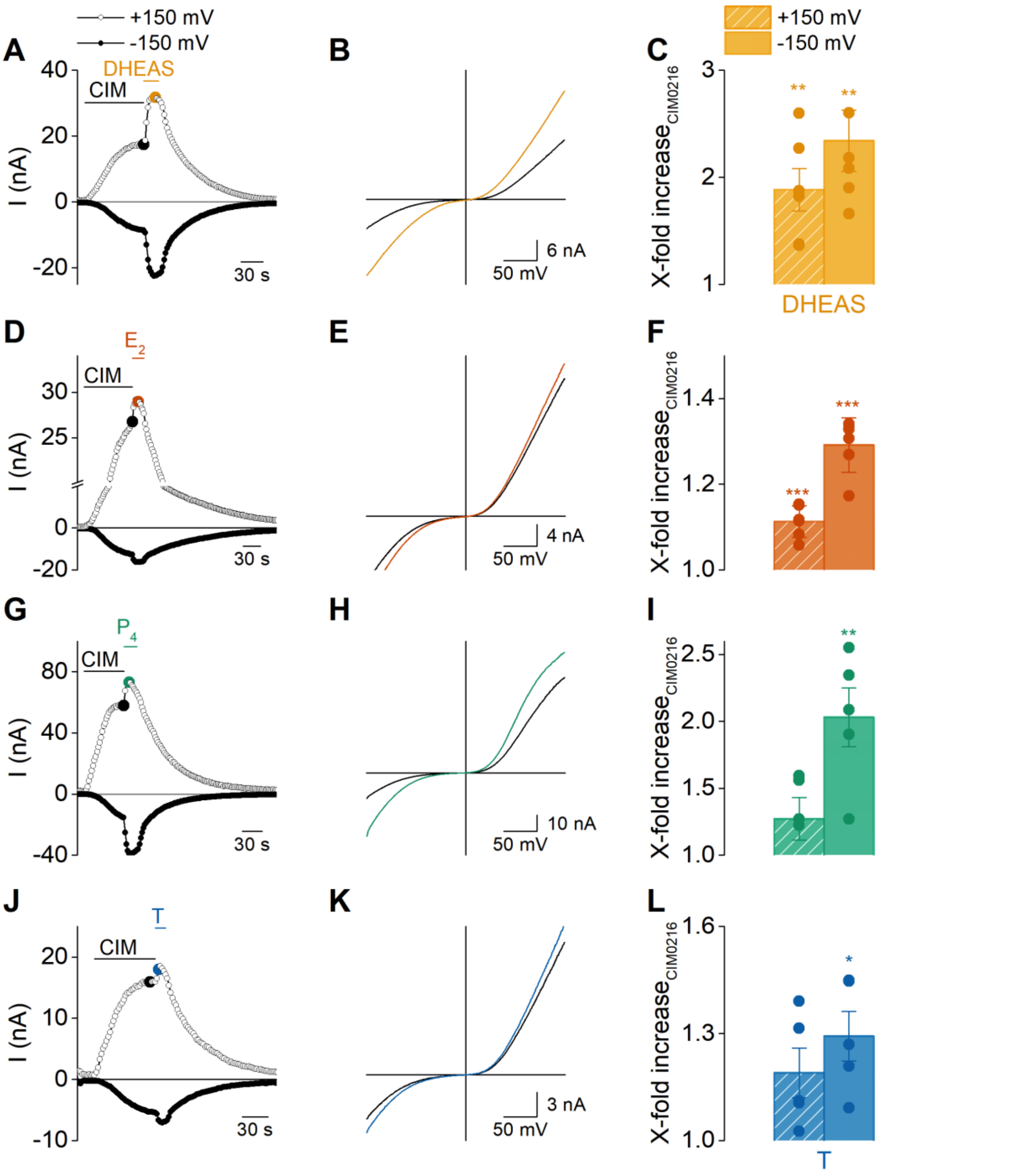

Figure S5. Potentiation of CIM0216 responses by DHEAS, estradiol, progesterone, and testosterone. (**A, D, G, J**) Time course of patch clamp experiments on HEK-TRPM3α2 cells upon application of 1 μM CIM0216, followed by an application of 100 μM DHEAS, E_2, P_4, and T, respectively. (**B, E, H, K**) The corresponding I-V curve of the indicated points in *(A, D, G, J)* after stimulation with DHEAS, E_2, P_4, and T, respectively. (**C, F, I, L**) X-fold potentiation of currents in presence of CIM0216 at -150 mV (shaded) and +150 mV for DHEAS (n = 6), E_2 (n = 6), P_4 (n = 5), and T (n = 5), respectively. Data are represented as mean ± SEM. Statistical analysis using a One Sample t-tests, p<0.05 *, p<0.01 **. P values for *(C):* +150 mV: p = 0.0068 and -150 mV: p = 0.0054; *(F):* +150 mV: p = 0.0007 and -150 mV: p = 0.0001; *(I):* +150 mV: p = 0.164 and -150 mV: p = 0.0094; *(L):* +150 mV: p = 0.052 and -150 mV: p = 0.0135.

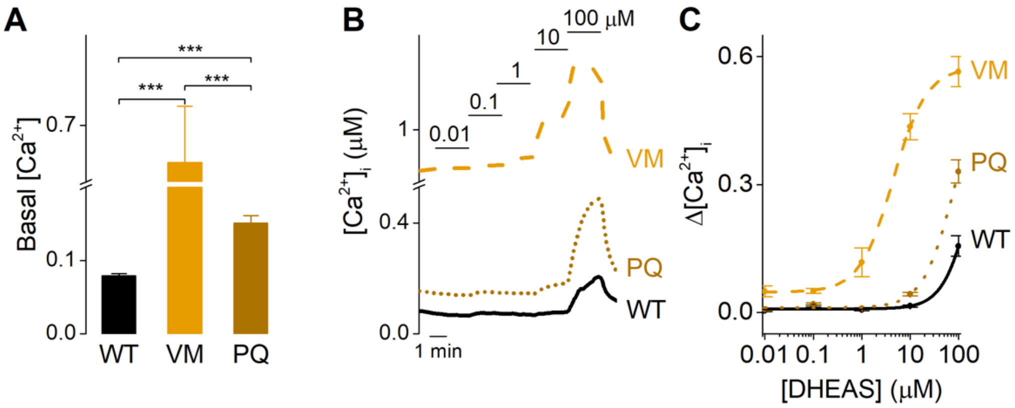

Figure S6. TRPM3 mutants showed an increased sensitivity to DHEAS. (**A**) Basal calcium concentrations of the V990M (VM) (N = 3, n = 314) and P1090Q (PQ) (N = 3, n = 294) TRPM3 variant are significantly increased compared to the wild type (WT) (N = 3, n = 184). (**B**) Time course of DHEAS concentration-response recordings in calcium imaging experiments on wild type (N = 3, n = 184), V990M (N = 3, n = 314) or P1090Q (N = 3, n = 294) HEK_hTRPM3YFP cells. (**C**) Concentration-response curves of DHEAS in wild type, V990M or P1090Q HEK_ hTRPM3YFP cells. Data are represented as mean ± SEM. Statistical analysis using a Kruskal-Wallis test, p<0.05 *, p<0.001 ***. P values for *(A):* WT/VM: $p < 1*10^{-15}$, WT/PQ: $p = 6.9*10^{-13}$, VM/PQ: $p = 1.5*10^{-7}$.
